# Self-management, care needs and clinical management of primiparous mothers during early labour – a qualitative content analysis

**DOI:** 10.1186/s12884-023-05453-4

**Published:** 2023-03-18

**Authors:** Antonia N. Mueller, Susanne Grylka-Baeschlin

**Affiliations:** Research Institute of Midwifery and Reproductive Health, School of Health Sciences, ZHAW Zurich University of Applied Sciences, Katharina-Sulzer-Platz 9, CH-8401 Winterthur, Switzerland

**Keywords:** Labour onset, Latent phase of labour, Early labour, Coping strategies, Needs assessment, Care needs, Labour management

## Abstract

**Background:**

Childbearing women face the problem of managing spontaneous onset of labour without professional support. It is their responsibility to diagnose and react to early labour and subsequently recognise the right time to seek support. Institutional guidelines of clinics aim to admit childbearing women when in established labour. This explains why women in early labour are often advised to stay at home, which can be overwhelming and dissatisfying. This study aims to understand the self- and clinical management of early labour and care needs of first-time mothers during early labour.

**Methods:**

A qualitative approach was used involving four focus group discussions with a total of *N* = 18 mothers. Included were primiparous women who had given birth at term within the last 6 months and who experienced spontaneous onset of labour. Elective caesarean section or induction of labour were thereby exclusion criteria. The interviews followed a semi-structured, literature-based guide. Content analysis was applied.

**Results:**

Thirteen codes were summarised within three themes: ‘self-management’, ‘care needs’ and ‘professional management’. Various coping strategies and measures such as positive thinking or taking a bath helped women in managing early labour at home. The need for reassurance, professional guidance and pain management led them to seek professional support, which was initially accompanied by a feeling of inhibition. This negative emotion was mostly unjustified since many women felt well cared for and taken seriously in their needs.

**Conclusion:**

Coping strategies and professional care help women going through early labour. Yet, there still exists insecurity about the justified timing in seeking professional support. An individual assessment of the women’s coping resources and their needs is required to promote shared decision making and give high-standard care.

## Background

Many women manage early labour with no or intermittent help of health professionals [1, 2], although the process of latent phase might have a great impact on the whole birth outcome [1, 3]. As part of the first stage of labour, the latent phase, also referred to early labour, is characterised by different symptoms such as contractions, watery fluid loss, sleeping disorders and emotional upheaval [4]. The process of labour during the latent phase is often slow, accompanied by varying levels of pain during the contractions [1] and little progress in cervical change [5]. There is still a lack of consensus on the definition of labour onset [5], as some describe it as the beginning of early labour while others refer to it as the beginning of the active first stage of labour with regular, painful contractions and cervical dilatation of three to five centimetres [6, 7]. The ambivalence of the definition of labour onset is not only reflected in the literature, but also in practice. Women face the problem of evaluating signs of onset of labour which often do not reflect midwives’ views of labour onset [4, 8]. Yet, diagnosing labour onset seems to be a benchmark for hospital admission [9].

Various approaches such as promoting comfort, distraction or receiving support and reassurance help women in dealing with early labour at home [10]. The success of such coping strategies seems to be dependent on the intensity and duration of the symptoms, supportive resources and the surrounding environment [10]. Nevertheless, early labour is often experienced by women and their partners with anxiety and uncertainty [2, 8, 9, 11, 12], which in addition to pain management [6, 11] are the main reasons to seek help and emotional support from health care professionals [9, 11, 13]. To react to such requests, Edmonds et al. [11] described the need of reassurance about the normality of the situation and the foetal wellbeing. Furthermore, knowledge about labour progress is reported as helpful to cope with the process and release insecurities [11, 14]. However, the timing of women’s needs to seek hospital admission in early labour is in conflict with institutional guidelines that recommend women to stay at home for as long as possible [6]. According to statements from midwives, the hospital was not found to be the best place to manage early labour [8, 15]. A major concern is the higher rate of medical interventions such as augmentation of labour or epidural analgesia in women presenting in early labour [3, 12, 16]. Therefore postponing hospital admission is believed to be in the women’s interests to prevent them from often unnecessary interventions [2]. Additionally, the high workload in the labour ward hinders midwives to allow women to be admitted in early labour [15, 17] and hospitalising women in established labour is more cost-effective for the institution [12, 18]. However, to support shared decision making it is essential to assess women’s coping mechanisms at home [9] and to understand their care needs in early labour [2, 8]. Although a series of measures to delay hospital admission such as home or telephone triage have been investigated, the delay of hospital admission has widely been experienced negatively by labouring women [2, 9]. Certainly, some women have claimed to feel empowered and encouraged in staying at home [9], while on the contrary Miller et al. [12] discussed a partial preference to being admitted to the hospital. The possibility of not being allowed into the hospital promotes insecurity in women since they face the challenge of recognising the right time to call the midwife [10] and fear of not being taken seriously [2]. In order to counteract these uncertainties, it can be helpful if the women and their accompanying person have already met the midwife in advance or are cared for by a known midwife as is the case, for example, in a midwife-led model of care [19, 20]. Women feel more empowered to manage early labour at home if they are given the possibility of having constant repeated contact with a midwife during this phase [21]. It is also easier for the midwives to notice the needs of women in early labour if the repeated assessment of the women’s wellbeing during latent phase of labour is done by the same midwife [15, 20].

The management of early labour of primiparous women remains challenging for women and health care professionals [8]. Several studies have investigated the experiences of early labour in the hospital setting [8, 9], but publications on such experience prior to hospital admission remain scarce [10]. Furthermore, a large number of studies have focused on the outcomes of clinical interventions during the latent phase of labour [7, 16] without addressing the individual opinion of labouring women receiving such support. It is therefore necessary to invest in research to better address the women’s self-management and the professional management of early labour and women’s subsequent care needs during this phase [14]. On the basis of this background, this study aims to understand the self-management and professional management of the latent phase of labour and care needs of first-time mothers during early labour.

## Methods

### Study design and setting

A qualitative study design was applied to understand the individual aspects of early labour management and care needs of first-time mothers [22]. The data was collected by focus group discussions from August 2021 to October 2021 in the German speaking part of Switzerland. The Standards for Reporting Qualitative Research (SRQR) guidelines were followed [23].

This publication is part of the larger GebStart-study, which aims to develop and validate a tool for advising first-time mothers during early labour [24]. Data from focus group discussions on early labour symptoms, management and care needs was collected to design the tool. Expectations about and perceptions of early labour symptoms will be published elsewhere since it would go beyond the scope of this article. This study therefore describes the qualitative findings on the management and care needs of women during early labour.

### Participants

Four focus group discussions with a total of *N* = 18 primiparous mothers were conducted using a purposive sampling method. Included in study participation were mothers of adult age (≥ 18 years) who had given birth within the last six months and for whom birth initiated spontaneously at term. Therefore, women with planned induction of labour, planned caesarean section or pre-term labour were not eligible for the study. The interviews were conducted in German, implying that knowledge of the German language was essential. There were no restrictions regarding birth setting, birth mode or model of care which explains the heterogeneity among the participating women. For instance, mothers who received care from a caseload midwife, meaning having one or a small group of independent midwives caring for them through pregnancy, childbirth and sometimes also the postpartum period, participated, but also women who gave birth in a birthing centre instead of a hospital. This supported a wide statement on experienced early labour and care needs during latent phase of labour. The targeted women were contacted by independent midwives who promote postpartum care in a home setting and by midwives that offer postnatal exercises. Women that expressed interest in participation were then contacted by one of the two researchers via telephone and informed about the study.

### Ethical considerations

Ethical approval for the GebStart-study was given by the Ethics Committees of Zurich and Ethics Committees of Northwestern and Central Switzerland (BASEC-Nr. 2021–00687) in July 2021. Oral and written information alongside a consent form were given and signed by all the participating women. Confidentiality and the ability to withdraw from the study without explanation were guaranteed at any time.

### Data collection

Focus group discussions were chosen as the method of data collection since it is a reasonable approach to receive information on people’s attitudes regarding the addressed topic [25]. The interaction of various individuals promotes an open discussion on a certain subject and supports the understanding of the diverse experiences of each participant [25]. A semi-structured interview guide was developed using evidence-based literature on the subject alongside the clinical experiences as midwives in the Swiss context of the two authors [7, 8, 13, 17, 26–28]. The following themes were addressed: experiencing early labour care, self-management in early labour and quality of care during early labour. Three of the interviews took place face-to-face in accordance with the current government regulations during the corona pandemic, meaning wearing face masks and maintaining a distance of 1.5 m from each other. One additional focus group discussion was held online. A follow-up one-to-one telephone interview with one participant was conducted since difficulties occurred with the internet connection during the online discussion and her statements seemed relevant for further understanding.

### Data analysis

Each interview was transcribed verbatim. Since Swiss German is not a written language, the interviews had to be translated to Standard German. To minimise any bias due to translation, transcription was performed to be most possibly accurate to the Swiss language. Therefore, the grammatical structure of the Swiss language was retained and terms without identical wording to Standard German were not translated. Subsequently, a content analysis was performed according to Mayring et al. [29]. A deductive and inductive coding was applied. The first coder analysed the first manuscript by applying an inductive and deductive coding strategy. The second coder reflected the performed analysis in a second step. Disagreements were then discussed, and consensus was found. Thereafter, all other manuscripts were coded in the same way as the first manuscript until consensus was found. Finally, a joint synthesis of the codes into themes was made. Expertise on qualitative research by additional researchers from the research team would have always been accessible if consensus on codes or themes had been problematic. Yet, agreement on the analysis did not need further counselling. The analysis was performed using Atlas.ti 9.

A forward translation method has been used where both authors translated the German citations into the English language [30]. Observed deviations were discussed and consensus was found. Finally, a native speaker English lecturer reviewed the translations.

## Results

A total of *N* = 18 primiparous women at term participated in the focus group discussions. Four different interviews were performed with five (27.8%) women in the first group, four women in the second and third group (22.2% each) and five (27.8%) in the fourth group, which was held online. Two women were included even though they did not meet the inclusion criteria. One woman reported to be at term, but during the interview it became clear that she had experienced a premature birth with 36 + 4 weeks of gestation. Additionally, another woman was included although her child was two weeks older than intentionally planned because she was not able to participate in the first group and arranged to participate in the online group, which was performed two months after the first interview. The exclusion of these participants post-study seemed unjustified as both women had already participated and focus group interviews are based on participants interaction [25]. Detailed information on demographic data of the participating women is shown in Table 1.

**Table 1:** Demographic data of participating women

Characteristics	Group 1	Group 2	Group 3	Group 4 online	Total
**Participants** (n (%))	5 (27.8)	4 (22.2)	4 (22.2)	5 (27.8)	18 (100.0)
**Age** (Mean (min-max))	31.4 (29–34)	31.5 (29–34)	32.0 (29–34)	34.0 (29–41)	32.3 (29–41)
**Marital status** (n (%))					
Married	3 (60.0)	4 (100.0)	1 (25.0)	2 (4.0)	10 (55.6)
Partner	1 (20.0)	0	3 (75.0)	3 (60.0)	7 (38.9)
Single	1 (20.0)	0	0	0	1 (5.6)
**Weeks of pregnancy** (min-max)	41–42	39–42	38–42	37–42	37–42
**Mode of birth** (n (%))					
Spontaneous Vaginal	5 (100.0)	3 (75.0)	3 (75.0)	3 (60.0)	14 (77.8)
Operative birth	0	1 (25.0)	1 25.0)	0	2 (11.1)
Section caesarea	0	0	0	2 (40.0)	2 (11.1)
**Model of care** (n (%))					
Standard	2 (40.0)	2 (50.0)	1 (25.0)	4 (80.0)	9 (50.0)
Midwife-led	2 (40.0)	1 (25.0)	3 (75.0)	0	5 (27.7)
Caseload midwife	1 (20.0)	1 (25.0)	0 (0.0)	1 (20.0)	4 (22.2)
**Place of birth** (n (%))					
Public hospital	3 (60.0)	3 (75.0)	4 (100.0)	5 (100.0)	15 (83.3)
Private hospital	0	1 (25.0)	0	0	1 (5.6)
Birthing centre	2 (40.0)	0	0	0	2 (11.1)
**Age of child in weeks** (Mean (min-max))	16.7 (7.7–25.0)	15.6 (4.0–22.5)	16.8 (8.0–23.7)	22.5 (13.1–28.6)	18.4 (4.0–28.6)

The participants reported several abnormalities during pregnancy and childbirth. Three women experienced premature contractions with cervical changes, another woman suffered from gestational diabetes, and one was socially stressed due to difficult family circumstances during pregnancy. Non-physiological aspects during childbirth were malposition of the foetal head such as persistent occiput posterior position, pathological foetal heartrate, high blood loss postpartum or placental retention leading to operative excision of the placenta.

Content analysis revealed the following three themes: ‘Self-management’, ‘care needs’ and ‘professional management’. Table 2 demonstrates a list of the 13 codes that have been summarised within the themes.

**Table 2:** Themes and corresponding codes emerging from the content analysis

Self-management	Care needs	Professional management
• Coping strategies	• Seeking support	• Telephone support
• Measures during early labour at home	• Inhibitions in seeking professional support	• Decision making regarding hospital admission
• Support by accompanying person	• Calling the midwife	• Midwifery care during early labour
	• Experiencing the decision regarding hospital admission	• Obstetric measures in early labour
	• Experiencing professional support	• Inclusion of the accompanying person

### Self-management

Women reported on how they coped when labour started and what measures they used during early labour while at home. When talking about coping strategies, it became clear, that women referred to it as the mental ability to guide themselves through this phase. One major coping strategy was described as believing the body’s ability to give birth and in trusting in the natural state of it. It was helpful to maintain strength by trying to relax and apply positive thinking.

*“I just truly believed in my body. I trusted in it that it can do that and that it also knows what to do. … and also, the baby instinctively knows what to do.” (Fo4F)*

*“I just always tried to focus on the fact that labour is not a pain, because you always imply pain with something negative. I've always said ‘look at it as a tool, because without these contractions, you won't have your son.’" (Fo2F)*

Different measures were applied to manage early labour at home. A few women mentioned that they felt the urge to move around or go for a walk. In contrast, several women mentioned that it was impossible for them to be active. They just needed a comfortable position and to focus on relaxation. Most women clearly emphasised the need for calmness. The mothers discussed that they wanted to keep some energy by trying to sleep, eat and drink properly, using different calming techniques such as massages by their partner or through aroma therapy. All women mentioned that they heard of the positive impact of taking a hot bath, which explains that most women tried this method at home. Using hot water through hot baths or by taking warm showers was regarded as helpful during early labour.

*“I have resolved, … , that I will definitely take a hot bath, firstly because it is said that you should have a bath to see if the contractions disappear or become more intense and secondly also, because I feel very good in water. I thought, this will definitely help me, … .” (Fo1A)*

*“I simply secluded myself in my room, I did not move around, but closed the shutters and lay sideways on the bed and listened to relaxing music, … .” (Fo3E)*

Other women mentioned that they tried to distract themselves by watching TV, concentrating on breathing or listening to music. In particular, those women who participated in a hypnobirthing antenatal course felt well guided by audiotapes that they got to know through the classes. Furthermore, women felt pleased having their partner or an accompanying person around. Some mothers mentioned that when labour started, they enjoyed some last moments of intimacy with their partner in the knowledge that they would not have time alone anymore. Others appreciated the great help of their partners during this phase of labour.

*“I was very happy that my husband was at home, and he was able to pack the last things and he also gave me a foot massage during the contractions, which was very good, ... and he had already started to track the contractions ....” (Fo1B)*

### Care needs

Women described a point during early labour where they felt that they did not want to be without professional support anymore. Women mentioned the need of reassurance of knowing the normality of the process. Another major need was to have someone to guide them. One woman explained that it was important for her that the midwife was not too passive, while another woman described that it was most helpful to have someone to give her clear instructions.

*“She also confirmed that everything is normal ..., and I thought that was good again, that you don't have to worry somehow.” (Fo3D)*

*“And then I was glad, she said 'now you're taking a bath and do this and that', I think she noticed that I couldn't really think about it anymore myself.” (Fo2B)*

Some reasons for seeking professional support were associated with receiving clinical interventions. Women wanted to gain information on the progress they are making during labour and on the health status of their child. In this context, having a vaginal check-up or auscultating the baby’s heartbeat was considered helpful to know the potential progress and normality of labour. Additionally, many women described a need to receive help in coping with the pain. After applying different analgesic measures at home, it was of interest to receive further pain killing methods under professional supervision either alternatively such as acupuncture, or pharmacologically.

“I just wanted to have real pain medication, … , because I didn’t want to try anything else anymore.” (Fo1A)

Most women who received standard care felt inhibitions in calling the midwife. On the one hand because they were scared that the midwife would not take them seriously, but on the other hand because they did not want to disturb the midwives.

*“I was quite nervous before the phone call because I / well I didn't know what to say, I think I said at the beginning, I think I'm in labour, but I don't know (laughter), yes, exactly, but I was also quite afraid that she [the midwife] would not take it seriously .” (Fo3C)*

Women emphasised a great relief when they received reassurance that their call was reasonable and when they felt they were being taken seriously. While some women hoped to be admitted to the hospital after calling, others clearly sought help for further advice on possible measures at home, or just wanted to inform the hospital about the situation. All the women appreciated the possibility of being able to repeatedly call again at any time, or even to go to the hospital when they did not feel comfortable at home anymore. Even more valued was a specific time for a call, regardless of whether the symptoms had subsided or not.

*“That actually felt good to know, ok, I have some guidance that I can follow and if I didn’t feel comfortable anymore, I could easily go and I would be welcomed … ” (Fo3E)*

Women emphasised that seeking help from the midwife was much easier if they already knew the midwife beforehand. A repeated exchange on the situation was more common in women who were cared for by a caseload midwife.

*"It really helped that the midwife already knew me. Um, simply from prenatal care and because I knew her personally beforehand, .... She knew what I needed now and what is simply good in this situation." (Fo2B)*

### Professional management

Many women felt very well cared for and emphasised a great satisfaction in the care they received. Most women who called the midwife because of contractions were advised to stay at home and given recommendations on measures to take at home. They were advised to call again if they felt unwell or the contractions seem to be progressing. Almost every woman was satisfied with this decision, yet for some women this strategy did not seem to be appropriate as they could not cope with the pain at home any longer.

*“Yes, why don't you go for a walk,' and I stood there thinking, 'Seriously? I'm not going for a walk now, I'm already screaming … , I'm not going outside where everyone can see me. What kind of stupid idea is that?'” (Fo1A)*

The advice regarding clinical admission was based on physical and emotional symptoms that the women mentioned on the phone or that the midwife could assess over the phone. Women who already seemed to have painful contractions or women whose amniotic membrane had ruptured were all advised to have at least a clinical check-up. Furthermore, it seemed that women who had already called during early labour were advised to come to the hospital/birthing centre for a check-up and possible admission.

*“She (the midwife) also said '... have breakfast at home and take a bath and see if it gets stronger and then call again at eight o'clock...'. And then I took bath, had breakfast and then I called again and then they said 'Yes, come over.'” (Fo1E)*

Many women mentioned that clinical measures in coping with early labour did not differ greatly to those measures that they applied at home. After admission in the clinic, women often went for a walk and tried different positions or took a bath and tried to relax. They received emotional support from a midwife, gained reassurance about their baby’s health through auscultation of the heartbeat and knowledge about labour progression by vaginal check-ups. Additionally medical interventions such as pharmacological analgesia such as opioids or epidural analgesia was administered. Also,medical labour inhibition was used meaning that any progress of labour was slowed down by stopping the contractions using tocolytic drugs for the women to get some rest.

*“They (midwives) took time for me and always checked for progressions and just asked what they could do to make it easier for me.” (Fo1A)*

*“And the epidural was then like a salvation, [ … ], the pain was gone and that helped so much.” (Fo4B)*

There were inconstancies in satisfaction with the care they received at the hospital. While some women stated that they were happy with the opportunity that a midwife was around although they did not need one-to-one care, others felt being second rank and left alone.

*“And afterwards I found it great, … , I feel like we’ve been alone most of the time, safe in this tub … . But it would have annoyed me if she kept talking to me all the time. I don’t know, maybe she noticed, but um, she just did what was necessary and that’s just been good for me.” (Fo3C)*

*“They told me ‘Yes, with these two to three centimetres, you are early' and so I did not have any priority.” (Fo1E)*

Moreover, health care professionals cared for the women’s partners by including them in the process, advising them how they could help their partner or by looking after them.

*Above all, they looked after our partners well, after my partner. So, I was always more afraid for him (laughs). He is not really a, how shall I say it, medical person. Or can't deal with it so well. I was always afraid he would keel over (group laughs). And she [the midwife] looked after him very well. (Fo3D)*

## Discussion

This study highlighted the subjective experience of managing early labour and the care needs of primiparous mothers. The main findings of this study showed that women already have a good knowledge about helpful coping strategies and measures that can be applied at home. The need for professional guidance and reassurance about the situation were major reasons to seek qualified support. Yet, there remained insecurities about the right time to call the midwives since women feared not being taken seriously.

Congruent with the findings of Beebe et al. (2006), this study showed that women had already figured out different strategies in coping with early labour at home. These were either applied intentionally or by using the knowledge gained at antenatal classes [10]. A focus on coping with early labour at home was based on maintaining energy through napping and eating. A calm atmosphere was considered helpful to rest. Yet, it seems that managing early labour at home was reaching a limit. In concordance with several previous studies, the major reasons for seeking professional help lay in uncertainty about the situation and in the need of clear professional guidance, such as advice on measures to apply or the need for medical interventions [9, 11]. The ability to cope with labour pain or fear and anxiety needed to be clearly assessed [8, 9]. For prehospital management, women were mostly guided to apply coping strategies that promote calmness. Only a few women felt comfortable in moving around at home which was different when in a hospital setting. Many participants of this study mentioned that they were encouraged to go for a walk or try different positions when managing early labour in the hospital setting. The potentially positive effect on labour progress by activity might be an explanation why women were more likely to be advised to be active while being in the hospital than at home [16]. Not only is it in the women’s interest to observe a progression in labour [11, 14], midwives also prefer women to be in established labour when admitted in the hospital [15].

The current findings showed the need for reassurance about the health of the child and the normality of the situation, which could to a certain extent only be given by a health care provider since expert opinion was favoured [11]. Hearing the foetal heartbeat through auscultation and having a midwife explain the process that was happening was needed for some women to be encouraged during early labour. Additionally, not only midwives, but also women relate to labour onset as being “the real thing” after progressive cervical dilation can be observed [2, 7]. Therefore, women wanted certainty about the progress of labour as measured by cervical dilation - an examination that a woman can hardly perform on her own [14].

The individually experienced intensity of the pain seemed to be a fundamental factor that makes analgesia desirable in early labour [2, 10]. In this study, women discussed that they would not have thought of trying to achieve pharmacological pain relief on their own at home since they did not feel comfortable in using analgesia without medical advice. None of the women mentioned that they were advised to take painkillers at home. Beebe et al. (2007) showed different results. According to their study, women used paracetamol at home autonomously to ease the pain [31]. Further research is needed to investigate if cultural differences exist in the advice and handling of taking pharmacological analgesia during early labour without medical supervision.

The decision to call the midwife remains challenging for many women [4] since they fear of not meeting the right timing or being rejected for admission in the clinic [2]. Nevertheless, in this study most women felt reassured and relieved after the first contact with a midwife. The majority stated that they were taken seriously and either empowered to try further coping strategies at home or were able to go to the clinic. As the results of this study show, the inhibition in calling the midwife could be lowered if the women already knew the midwife or the institution beforehand and emphasised the positive effect of dealing with early labour at home if they were in a constant exchange with a midwife. Lowering uncertainties in seeking professional support can possibly be facilitated by applying midwife-led care models where women already get to know the midwife in pregnancy and have better access to contact the midwife at labour onset [19, 20].

### Strengths and limitations

Due to its qualitative design, this study gives an in-depth insight into the process of self-management, identification of care needs and clinical care of labouring women in early labour. The usage of focus group discussions underpins the individual aspects of the phenomenon [25] while the heterogenous sample of 18 primiparous mothers allows a fair statement on the various experiences of coping with early labour and their reasons for seeking professional support. Different studies have focused on the management of early labour after hospital admission [8, 17]. Yet, the focus of literature on coping strategies of labouring women during early labour at home is scarce and outdated. Therefore, this study aids the understanding of a perpetually challenging phenomenon in practice.

Nevertheless, this study is prone to some limitations. The four very different discussions suggest that data saturation has not been achieved. The study sample represents women with a rather high educational level. Such selection bias is not uncommon in health research [32, 33], but can be argued that transferability is of higher focus in qualitative research than generalisability [33]. The interviews were all held in Swiss German and subsequently transcribed into high German. Such translation has a tendency that certain analytical processes are already being performed during transcription [34]. Participant’s quotes have been translated to English to enable the global academic community to understand the findings of this research [35]. Yet, such translation might jeopardise the validity of the results [35]. To minimise such issues a forward translation from German to English has been performed independently by the two researchers and has additionally been proofread and discussed by a native English speaker. Furthermore, the lack of a possible recall bias due to the late response of the study after the women have experienced early labour cannot be guaranteed.

## Conclusion

Finally, it can be said that many women feel comfortable with the care they receive during early labour. Antenatal classes prepare women with coping strategies at home and in understanding reasons of postponing clinical admission [10, 13]. Yet, it remains a difficult task for women to evaluate the right timing in seeking professional help [1]. This is often accompanied by a feeling of inhibition in calling the midwife for women who have not had any previous contact with the midwife. This negative emotion often remained unjustified since the majority received the care they wished for.

The intention of preventing women from unnecessary interventions by postponing clinical admission requires discussion since it might not meet childbearing women’s needs [9, 13] and since its outcomes still remain unclear [7]. An individual assessment of the women’s coping resources and their needs is required to firstly promote shared decision making and secondly give high-standard care.

## Data Availability

Data is available on reasonable request by the authors.
